# Arrhythmias in Patients with Congenital Heart Disease: An Ongoing Morbidity

**DOI:** 10.3390/jcm12227020

**Published:** 2023-11-10

**Authors:** Despoina Ntiloudi, Spyridon Rammos, Maria Karakosta, Alkistis Kalesi, Nearchos Kasinos, George Giannakoulas

**Affiliations:** 1 Department of Cardiology, Tzaneio General Hospital of Piraeus, 185 36 Piraeus, Greece; mariakarakosta@hotmail.com (M.K.); eileen_calessis@yahoo.gr (A.K.); nearchoskassinos@gmail.com (N.K.); 2 Echocardiography Training Center of Tzaneio ‘D. Beldekos’, 185 36 Piraeus, Greece; 3 Department of Pediatric Cardiology and Adult Congenital Heart Disease, “Onassis” Cardiac Surgery Center, 176 74 Athens, Greece; srammos@gmail.com; 4 First Department of Cardiology, AHEPA University Hospital, Aristotle University of Thessaloniki, 546 36 Thessaloniki, Greece; g.giannakoulas@gmail.com

**Keywords:** arrhythmia, congenital heart disease, sudden cardiac death, ablation

## Abstract

With the aging of congenital heart disease (CHD) patients, the burden of arrhythmias is expanding. Atrial arrhythmias, especially intra-atrial reentrant tachycardia and atrial fibrillation, are the most prevalent forms of arrhythmia. Managing comorbidities, such as obesity, using pharmacotherapy, including antiarrhythmics and anticoagulants, and ablation therapy has become the cornerstone of arrhythmia management. Ventricular tachycardias are also not rare; however, except for tetralogy of Fallot patients, recommendations for the use of implantable cardioverter defibrillators for primary prevention in other CHD patients are still not well established. Patients with CHD might also present with atrioventricular blockages because of their anatomy or following a surgical procedure. The scope of this article is to review the current knowledge and discuss the future directions regarding arrhythmia management in CHD patients.

## 1. Introduction

**Figure 1:**
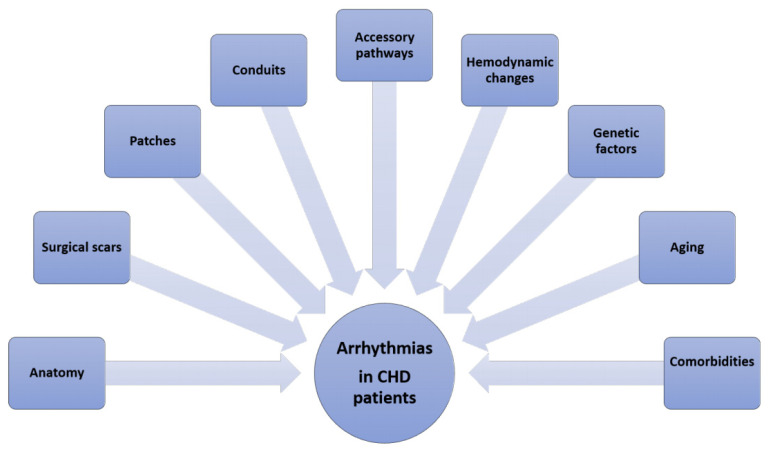
The etiology of arrhythmia presentation in patients with congenital heart disease. CHD: congenital heart disease.

The number of patients with congenital heart disease (CHD) is constantly growing, and throughout their lifespan, they might encounter complications related to their cardiac anatomy or the underlying surgical or percutaneous procedures [1]. The high burden of arrhythmias in this patient population is well established [2,3]. CHD patients face increasing hospitalization rates due to arrhythmic episodes, and arrhythmia is the leading cause of unscheduled hospitalization [4,5,6]. Arrhythmias have been associated with syncope, heart failure, thromboembolic events and sudden death [7]. The etiology can vary, including the underlying CHD anatomy, displaced or malformed conduction systems, surgical scars, patches, conduits, hypoxemia, long-standing hemodynamic changes and genetic factors (Figure 1). The scope of this article is to review the types of arrhythmias in the CHD patient population, including atrial, ventricular and bradyarrhythmia, the management options for arrhythmias, and the future perspectives in this field. 

## 2. Atrial Arrhythmias

Atrial arrhythmias are very common, especially in older CHD patients. In a retrospective study of 38,428 patients, 15% had a history of atrial arrhythmia [7]. In this study, the probability of atrial arrhythmia development was calculated as 7% and 38% in patients aged 20 and 50 years old, respectively. The presence of atrial tachycardias has been related to complications such as heart failure, stroke and mortality [8]. At a median 6.5 years follow-up of 14,224 CHD patients, those with a history of atrial arrhythmia had a 2-fold increased risk of death and a 4-fold increased risk of heart failure-related hospitalizations, while stroke risk was increased with new-onset atrial arrhythmia. 

A histopathology study demonstrated significant right atrial remodeling (extent of fibrosis, myocyte diameter, capillary distance and CD45-positive cell infiltration) in samples obtained from CHD patients with a history of atrial arrhythmia, followed by patients with CHD without a history of atrial arrhythmia and then the controls [9]. In contrast to the controls, all of the parameters of right atrial remodeling were related to age and therefore the duration of volume and/or pressure overload in CHD patients. Overload of the right atrium can be detected in several CHD subtypes, such as in longstanding left-to-right shunt in atrial septal defect patients and in tricuspid regurgitation [10]. 

Among atrial tachycardias, intra-atrial reentrant tachycardia (IART) is the most common atrial arrhythmia, with significantly higher rates in patients with complex CHD [11]. The incidence of IART is highest in patients with atrial switch for the transposition of great arteries (i.e., Mustard/Senning procedures) and patients with Fontan circulation, especially those with an atriopulmonary Fontan operation given the systematic atrial dilatation [11,12]. These arrhythmias are associated with general re-entry mechanisms [13]. Due to surgical scars and patches, slow conduction areas and conduction blocks are common in these patient populations. Slow intra-atrial conduction in these cases results in a higher likelihood of cavotricuspid isthmus (CTI)-related IART compared to the general population [14]. Nevertheless, CHD patients also frequently experience non-CTI-related IART, which is linked to surgical patch areas and fibrotic or scarred regions. Depending on the type of CHD, the proportion of non-CTI-related IART can range from 50% to 75% [15,16]. Patients with complex CHD have a higher incidence of both types of IART due to a greater number of surgical scars, which create areas of slow conduction, and residual lesions that result in pressure and atrial overload, diminishing intra-atrial conduction [17]. Specific information regarding circuit locations and their relationship with atrial scarring is limited. Patients with an Ebstein anomaly and those with congenitally corrected transposition of the great arteries and systematic atrioventricular valve with Ebstein valve characteristics often have accessory pathways, and therefore may present with atrioventricular re-entrant tachycardia [10]. Patients with an Ebstein anomaly and pre-excitation in their electrocardiogram may have more than one accessory pathway, which is associated with an increased risk of sudden cardiac death [18]. Atrial flutter may also be detected in CHD patients, especially those with a dilated right atrium, such as unoperated atrial septal defect patients [2].

Atrial fibrillation (AF) is the second most prevalent atrial tachycardia following IART [11]. In addition to the congenital heart disease substrate and older age, cardiovascular comorbidities such as systemic arterial hypertension, diabetes mellitus and obesity are associated with AF in this patient group [10]. In contrast with IART, which increases in prevalence in parallel with CHD complexity, AF increases as the population ages [11]. However, AF in the CHD population is reported in younger ages compared to the general population and AF episodes can coexist with regular atrial tachycardia [19]. The arrhythmia pattern changes progressively from paroxysmal to persistent or prevalent [11,19]. AF is common among patients with atrial switch for the transposition of the great arteries, Fontan circulation, tetralogy of Fallot (TOF), atrioventricular septal defect and atrial septal defect [20]. Regarding atrial septal defect patients, about 10% of patients who have not undergone closure present with atrial arrhythmia, and patients older than 40 years of age more frequently have AF [20,21,22]. Percutaneous or surgical atrial septal defect closure prevents this outcome, especially when performed at an early age [20,21,22]. Following atrial septal defect closure, the incidence of new-onset AF is relatively low, and it was reported to be 1.8 patients per 100-patient-years at follow up; however, it reached 5.2 patients per 100-patient-years among patients aged more than 60 years old [23]. It is interesting that in a recent metanalysis regarding AF development after patent foramen ovale closure, AF mainly developed in the first 45 days [24]. Likewise, in the general population, AF is associated with an increased risk of stroke and heart failure, and it should be noted that atrial contraction contributes about 20% of the systemic ventricular stroke volume at rest [10]. 

The management of atrial arrhythmias is of high importance given that these patients are often of a young age and have normal atrioventricular node function, and therefore a rapid atrial tachycardia can lead to syncope or even sudden cardiac death in patients with systemic ventricular dysfunction, especially in those with a systemic right ventricle [10]. In the current era, the correlation between the success rates of AF ablation and the complexity of CHD remains a controversial issue. The ablation of atrial septal defects seems to have the highest success rates [25]. A meta-analysis published at 2019 by Pranata R. et al. showed no great differences between simple–moderate and complex CHD, with the latter needing more advanced equipment and being performed by experts in tertiary centers [25]. However, repeat ablation procedures can improve the outcomes. In the same systematic review and meta-analysis about catheter ablation of AF in CHD, the one-year AF freedom rates varied from 32.8% to 63%, with female gender, anatomic complexity, persistent AF, and larger left atrial dimensions being associated with a higher risk of AF recurrence [25]. Pulmonary vein isolation is considered the cornerstone of ablation strategies, with several different approaches such as substrate modification, left atrial appendage isolation, left atrial posterior wall isolation and superior vena cava isolation in patients who experience AF recurrences receiving interest [26]. The emerging role of artificial intelligence in assisting ablation procedures and predicting AF recurrences in this heterogeneous population should be further investigated [27]. In general, ablation therapy and the Maze procedure in CHD patients have good results in maintaining sinus rhythm, and given the side effects arising from the long-term use of most antiarrhythmic drugs, in the European Society of Cardiology Guidelines for adults with CHD, catheter ablation is recommended over long-term medical therapy for symptomatic sustained SVT in patients with mild CHD with an indication level class I [28]. In patients with moderate and severe CHD, catheter ablation should be considered for symptomatic sustained SVT, or if SVT is potentially related to sudden cardiac death (patients with an Ebstein anomaly, atrial switch for TGA, ccTGA and Fontan patients) [28]. In a prospective single-center study, ablation of IART had a success rate for maintaining sinus rhythm of 78% [29]. The predictors of a recurrence of arrhythmia were non-cavotricuspid-isthmus-related IART, a long PR interval and previous or induced AF. These three predictors consisted of a risk score identifying three levels of recurrence risk: 5.8%, 20%, and 58.5%. The success rates of AF ablation vary based on the complexity of CHD, with more complex cases generally having lower success rates. 

Anticoagulation is recommended in adult patients with moderate or complex CHD and persistent or permanent AF/IART, while patients with thromboembolic risk and mild CHD should be assessed using the CHA2DS2-VASc score [28]. Non-vitamin K oral anticoagulants seem to be safe and effective in the CHD population [30,31]. However, data on patients with Fontan circulation and cyanotic heart disease are insufficient [32,33]. In patients with mechanical valve or mitral valve stenosis, non-vitamin K oral anticoagulants are contraindicated, similarly to the general population [32]. 

## 3. Ventricular Arrhythmias

Premature ventricular contractions, non-sustained ventricular tachycardia and sustained ventricular tachycardia (monomorphic, polymorphic and ventricular fibrillation) are all forms of ventricular arrhythmias. Ventricular ectopy is common in adults with CHD. Polymorphic ventricular tachycardia and ventricular fibrillation are associated with extensive myocardial hypertrophy, diffuse fibrosis, myocardial ischemia and/or severe systemic ventricular dysfunction [3]. Non-sustained ventricular arrhythmias are not uncommon in CHD patients, but they are often asymptomatic and are detected in Holter or device monitoring [2]. Ventricular tachycardias are widely reported in TOF patients, where monomorphic ventricular arrhythmias are the result of ventriculotomy or a scar in right ventricle outflow track, but may be documented in a wide spectrum of CHD substrates [3,34]. In a multicenter cohort study in patients with transposition of the great arteries, half of the patients presented with monomorphic ventricular tachycardia, 34% presented with polymorphic ventricular tachycardia and 17% presented with ventricular fibrillation [35]. 

All CHD patients presenting with ventricular tachycardia should be also investigated for mechanisms other than their CHD substrate, such as coronary artery disease, ventricular dysfunction, long QT syndrome, arrhythmogenic cardiomyopathy or other syndromes related to malignant arrhythmias [2]. According to the ESC Guidelines, when recurrent monomorphic VT, incessant VT, or electrical storm are not managed through medical therapy, catheter ablation is indicated as an adjunctive therapy to ICDs. In a systematic review of catheter ablation therapy in patients with TOF and VT, repeat catheter ablation was required in less than one-fifth of the cases, the in-hospital mortality rate was zero, the mortality rate over a 6-year follow up period was 3%, and the need for antiarrhythmic drugs was substantially reduced after the procedure [36]. Specifically, amiodarone and β-blocker use was reduced from 40% to 20% and from 58% to 28%, respectively [36]. Surgical cryoablation may also be considered during pulmonary valve replacement in patients with clinical VT [37].

## 4. Sudden Cardiac Death

Sudden death is among the most prevalent causes of death in the CHD population [38,39]. Even though the incidence of sudden death in CHD patients is low, between 0.09% and 0.26% annually, it is much higher compared to the aged-matched general population [40,41]. In most cases, sudden cardiac death is the outcome of ventricular arrhythmia, and in less than 5%, the cause is an atrioventricular blockage [10]. In a metanalysis of 2162 CHD patients with an implantable cardioverter defibrillator (ICD) who were followed for 3.7 years, 24% received one or more appropriate antitachycardia pacing or shock (22% of patients with primary prevention and 35% of secondary prevention) [42]. However, one-fourth of the patients had an inappropriate shock and more than one-fourth had a complication related to lead placement [42]. The all-cause mortality was 10%, lower than the conventional ICD population [42,43].

The small number of patients and the heterogeneity of CHD limit the development of predictive models for sudden cardiac death. For instance, induced sustained ventricular tachycardia is a predictive factor of ventricular tachycardia for patients with TOF, but not those with transposition of the great arteries [34,35]. In patients with transposition of the great arteries and atrial switch surgery, sudden cardiac death was associated with the presence of symptoms of arrhythmia or heart failure at the most recent follow up and a history of documented atrial flutter or AF, while in another study, severe systemic atrioventricular valve regurgitation and/or systemic ventricle dysfunction were significant risk factors for sudden death [44,45]. 

Regarding TOF patients, a risk score for appropriate ICD shocks was developed from a retrospective multicenter study, including prior palliative shunt (2 points attributed), inducible sustained ventricular tachycardia (2 points), QRS width ≥ 180 ms (1 point), ventriculotomy (2 points), non-sustained ventricular tachycardia (2 points) and left ventricle end diastolic pressure ≥ 12 mmHg (3 points) [34]. According to this score, TOF patients are stratified in three categories: low risk (risk score 0–2), intermediate (risk score 3–5) and high risk (risk score 6–12), with annualized rates of appropriate shocks of 0%, 3.8% and 17.5%, respectively.

Recently, the PREVENTION-ACHD risk score was developed and studied prospectively in adults with CHD, consisting of coronary artery disease, New York Heart Association class II/III, supraventricular tachycardia, systemic ejection fraction < 40%, subpulmonary ejection fraction < 40%, QRS duration ≥ 120 ms, and QT dispersion ≥ 70 ms, and showed relatively satisfactory results in assessing sudden cardiac death or ventricular tachycardia/fibrillation risk [46,47].

Given the young age and the low death rate of CHD patients, the cumulative beneficial effect of ICD implantation will be greater for this patient group. However, high rates of inappropriate shocks and complications related to lead implantation should be taken into account when assessing a patient for ICD implantation [42]. A reversible cause of aborted cardiac arrest due to VF should be excluded before implanting an ICD (Level of recommendation I) [28]. Hemodynamic evaluation and repair of a significant lesion when indicated are also mandatory. When a biventricular physiology is present in CHD patients, the extrapolation of general recommendations indicates ICD implantation in symptomatic heart failure patients (New York Heart Association II/III) with an ejection fraction of the systemic ventricle less than 35%, who have received for more than 3 months of optimal medical therapy, provided that they have a good clinical status with a life expectancy of more than a year (Level of Evidence IIa). In TOF patients, ICD implantation for primary prevention should be considered based on a personalized approach and taking into account the aforementioned risk scores and factors. For TGA patients, given that the data are sparse, non-sustained VT, NYHA II/III, a severe atrioventricular valve bundle branch block QRS morphology, and a QRS ≥ 140 ms may be taken into account according to the guidelines. Finally, in the case of a syncopal episode probably attributed to arrhythmia and either advanced ventricular dysfunction or inducible VT/VF upon programmed electrical stimulation, ICD implantation should be considered.

## 5. Bradyarrhythmias

In patients with CHD, sinus node dysfunction may arise as a complication following a procedure because of injury either directly to the sinus node or its artery, such as Mustard or Senning atrial switch or the Fontan procedure [2]. Patients with a left atrial isomerism lack a sinus node and therefore typically present with a low atrial or junctional rhythm.

Atrioventricular blockages can also be caused by the CHD substrate or the CHD surgical procedure [10]. Atrioventricular blockages can be observed in patients with congenital AV blockages but also in patients with congenitally corrected transposition of the great arteries, atrioventricular septal defects, heterotaxy syndrome, a single ventricle with L-looping and atrial septal defects. Postoperatively, atrioventricular blockages can be seen in ventricular septal closure, atrioventricular septal defect repair, tetralogy of Fallot repair, congenitally corrected transposition of the great arteries and left ventricle outflow track surgeries. The risk of complete atrioventricular blockage in patients with congenitally corrected transposition of the great arteries is 2% per year [10]. As far as ventricular septal defects are concerned, post-procedural conduction disturbances seem to be higher during the transcatheter closure of perimembranous VSD. This is mainly attributed to the proximity of the atrioventricular conduction system to the rims of the defect, particularly the remnants of the membranous septum [48]. The use of the eccentric Amplatzer occluder, originally derived from the occluder used to seal muscular defects, initially showed promising results with minimal complications [49]. However, larger studies have highlighted the emergence of significant conduction disturbances, such as the acute or long-term development of complete heart blockages, as serious complications, resulting in a gradual decrease in its use [48,50].

Pacemaker implantation should be considered when ablation fails or is not possible in adults with CHD and bradycardia–tachycardia syndrome to prevent IART. In severe CHD and sinus junctional bradycardia (daytime heart rate < 40 beats per minute or pauses > 3 s) and in patients with CHD and compromised hemodynamics due to sinus bradycardia or loss of AV synchrony, permanent pacing should also be considered.

## 6. Cardiac Resynchronization Therapy (CRT)

Interventricular and intraventricular electromechanical dyssynchrony, namely discoordination in contraction due to electrical activation delay, can lead to pathologic ventricular remodeling and dyssynchronous heart failure [10]. Especially in patients with systematic left ventricle and left bundle branch blockages or right ventricular pacing, left ventricle free wall and base depolarization and contraction are significantly delayed compared to those of the right ventricle, interventricular septum and the apex. Consequently, when one wall contracts, the other is stretched in the opposite direction, leading to insufficient left ventricle contraction and more specifically to increased left ventricle systolic volume and reduced stroke volume. Therefore, a part of myocardial work is wasted. 

In adults with CHD, conventional ventricular pacing rather than a bundle branch blockage is the major cause of systemic ventricular dyssynchrony [51]. CRT can be a therapeutic option for patients with heart failure undergoing optimal medical treatment and ventricular dyssynchrony. According to the European Society of Cardiology position paper on arrhythmias in CHD patients, CRT is indicated for patients with a systemic left ventricle with an ejection fraction ≤ 35%, a sinus rhythm, a wide QRS complex > 150 ms with a complete left bundle branch block QRS morphology (spontaneous or paced), and NYHA functional Class II—ambulatory IV, while in those with the same characteristics and QRS complex 120–149 ms or a QRS > 150 ms and a complete right bundle branch blockage, CRT implantation can be useful [28]. CRT is also indicated in those who have a systemic ejection fraction ≤ 35%, a narrow QRS complex, and NYHA functional Class II—ambulatory IV, who are undergoing new device placement or replacement with an anticipated requirement for >40% ventricular pacing. CRT is not indicated in patients with a narrow complex QRS < 120 ms and those who are expected to survive with good functional capacity for less than a year.

In patients with a systematic right ventricle, CRT can be useful in patients with an ejection fraction < 35%, NYHA functional Class II ambulatory IV and a wide QRS complex > 150 ms with a complete right bundle branch blockage QRS morphology (spontaneous or paced), and may be considered in those with a QRS complex of 120–150 ms [28]. In a CHD cohort study, 9.3% of patients with transposition of the great arteries and 6.1% of patients with congenitally corrected transposition of the great arteries were considered eligible for CRT [52]. As far as patients with a single ventricle are concerned, CRT can be useful for patients with a single ventricle with an ejection fraction < 35%, NYHA functional Class II—ambulatory IV and a wide QRS complex > 150 ms due to intraventricular conduction delay causing either a complete right or left bundle branch blockage QRS morphology and may be considered for those with a QRS complex of 120–150 ms [28].

Long-term data are lacking, and the efficacy of CRT in CHD patients may vary across defects and depend on the individual anatomy and causes of dyssynchrony [53]. It should be noted that anatomical constraints often necessitate thoracotomy or hybrid transcatheter–surgical lead implantation. In patients with atrial switch, a hybrid approach is commonly used, while in single-ventricle patients, non-transvenous lead implantation is mainly required. Septal or His pacing may decrease pacing-associated dyssynchronopathy among CHD patients. Multicenter prospective collaborative efforts should be encouraged.

## 7. Discussion

The management of arrhythmias in patients with CHD is a major challenge and affects their prognosis as well as their quality of life. Patients with moderate or complex CHD lesions and documented arrhythmia must be referred to CHD centers with a multidisciplinary team and expertise in the assessment and management of arrhythmias related to CHD. Patients with transposition of the great arteries and atrial switch operation have particularly abnormal hemodynamic responses to increased heart rates, and those with atrial tachyarrhythmias are susceptible to degeneration to ventricular tachyarrhythmias [18]. In Fontan patients, even though the rate of atrial tachyarrhythmia has markedly decreased in patients with total cavopulmonary connection compared to the older atriopulmonary connection, it still afflicts 10% to 15% of Fontan patients within a 15-year period. In these patients, atrial tachyarrhythmias are poorly tolerated [6,18]. In Eisenmenger patients, right atrial dilatation and remodeling predispose to atrial tachyarrhythmias, which are also poorly tolerated in this patient group. 

All patients should be checked for reversible causes of arrhythmia, including hyperthyroidism, inflammation as well as going through a hemodynamic assessment for residual or new hemodynamic abnormalities [28]. Maintenance of the sinus rhythm is important for all CHD patients. In cases of hemodynamic instability, cardioversion is indicated, regardless of the anticoagulation status. For atrial arrhythmias, if there is no hemodynamic instability and the arrhythmia is well tolerated, cardioversion can be performed following the general guidelines: appropriate anticoagulation for 3 weeks or transesophageal echocardiography to rule out thrombus. 

In cases where catheter ablation fails or is not possible, amiodarone may be considered to prevent atrial tachycardia/ AF recurrence, especially in patients with systemic ventricular dysfunction, hypertrophy of the systemic ventricle, or coronary artery disease [28]. Amiodarone should be used with caution in patients with cyanotic CHD, a low body weight (<21 kg/m^2^), hepatic, thyroid, or pulmonary disease or prolonged QT interval. Nevertheless, long-term administration is limited by the frequent side effects, especially in these relatively young patients. In general, the early recognition and management of arrhythmias in CHD patients is essential for long-term therapy success, given that arrhythmia burden progresses gradually from paroxysmal to permanent [32].

It is also important before any surgical or percutaneous procedure in CHD patients to assess their arrhythmiological substrate. In particular, patients with an Ebstein anomaly, given the high rate of accessory pathways in these patients, usually undergo an electrophysiological study before surgical procedure. Patients with TOF should also undergo an electrophysiological study before transcatheter pulmonary valve implantation. 

## 8. Conclusions

Arrhythmia is common in the CHD population, and considering the unique anatomic and hemodynamic features of these patients, its management is challenging. Advances in the field have majorly contributed to treating these patients, and future perspectives are anticipated to help further. 

## 9. Future Directions

Advances in techniques and technology have contributed to arrhythmia management in this unique patient population, and remote workup has contributed even further. However, still there are fields to improve in order to move forward in this direction. 

Catheter ablation outcomes can be improved by further understanding of the underlying mechanisms and substrates. Further developments in mapping technology can be very useful in the identification of arrhythmia substrates.

The indications of ICD implantation are still a major challenge in CHD patients. In spite of the numerous cohort studies, there are no randomized control trials in the field that could help refine and validate risk stratification in CHD patients. In particular, in patients with a single ventricle and those with a systemic right ventricle, there is lack of prediction models for ICD implantation.

Leadless pacing systems are very useful in patients in whom access to the subpulmonary ventricle from the venous system is anatomically impossible. However, these systems can currently sense and pace only the ventricular myocardium, limiting their application.

With the advancement of technology, wearable devices are becoming popular, and arrhythmias are more easily detected. However, there are no available data yet on whether the arrhythmias detected by these devices have clinical implications and should be treated accordingly.
